# Supplementary material for: Pyruvate Dehydrogenase Kinase 1 inhibition mediated oxidative phosphorylation enhancement in cartilage promotes osteoarthritis progression
Source: BMC Musculoskelet Disord. 2023 Jul 20;24:597. doi: 10.1186/s12891-023-06585-6 (PMC10357736; doi:10.1186/s12891-023-06585-6)
Supplement: Supplementary file 1 — Additional file 1. [file 12891_2023_6585_MOESM1_ESM.zip › MF.Gsea.1653627316242/GOMF_O_METHYLTRANSFERASE_ACTIVITY.html]

Details for gene set GOMF\_O\_METHYLTRANSFERASE\_ACTIVITY[GSEA]

|  || Dataset | 55457\_collapsed\_to\_symbols.cls.cls #OA\_versus\_normal.cls.cls #OA\_versus\_normal\_repos |
| Phenotype | cls.cls#OA\_versus\_normal\_repos |
| Upregulated in class | OA |
| GeneSet | GOMF\_O\_METHYLTRANSFERASE\_ACTIVITY |
| Enrichment Score (ES) | 0.51313007 |
| Normalized Enrichment Score (NES) | 1.5992963 |
| Nominal p-value | 0.03448276 |
| FDR q-value | 0.13990901 |
| FWER p-Value | 0.984 |
Table: GSEA Results Summary

  

Fig 1: Enrichment plot: GOMF\_O\_METHYLTRANSFERASE\_ACTIVITY      
 Profile of the Running ES Score & Positions of GeneSet Members on the Rank Ordered List

  

| SYMBOL | TITLE | RANK IN GENE LIST | RANK METRIC SCORE | RUNNING ES | CORE ENRICHMENT || 1 | COMT | catechol-O-methyltransferase [Source:HGNC Symbol;Acc:HGNC:2228] | 335 | 0.732 | 0.1028 | Yes |
| 2 | LCMT1 | leucine carboxyl methyltransferase 1 [Source:HGNC Symbol;Acc:HGNC:17557] | 735 | 0.585 | 0.1746 | Yes |
| 3 | ARMT1 | acidic residue methyltransferase 1 [Source:HGNC Symbol;Acc:HGNC:17872] | 1080 | 0.500 | 0.2359 | Yes |
| 4 | ICMT | isoprenylcysteine carboxyl methyltransferase [Source:HGNC Symbol;Acc:HGNC:5350] | 1140 | 0.488 | 0.3171 | Yes |
| 5 | ASMTL | acetylserotonin O-methyltransferase like [Source:HGNC Symbol;Acc:HGNC:751] | 1143 | 0.487 | 0.4027 | Yes |
| 6 | MRM2 | mitochondrial rRNA methyltransferase 2 [Source:HGNC Symbol;Acc:HGNC:16352] | 1880 | 0.375 | 0.4115 | Yes |
| 7 | COQ3 | "coenzyme Q3, methyltransferase [Source:HGNC Symbol;Acc:HGNC:18175]" | 2164 | 0.342 | 0.4497 | Yes |
| 8 | MEPCE | methylphosphate capping enzyme [Source:HGNC Symbol;Acc:HGNC:20247] | 2554 | 0.297 | 0.4717 | Yes |
| 9 | FTSJ1 | FtsJ RNA 2'-O-methyltransferase 1 [Source:HGNC Symbol;Acc:HGNC:13254] | 2664 | 0.284 | 0.5131 | Yes |
| 10 | PCMT1 | protein-L-isoaspartate (D-aspartate) O-methyltransferase [Source:HGNC Symbol;Acc:HGNC:8728] | 3925 | 0.162 | 0.4439 | No |
| 11 | PCMTD2 | protein-L-isoaspartate (D-aspartate) O-methyltransferase domain containing 2 [Source:HGNC Symbol;Acc:HGNC:15882] | 3975 | 0.158 | 0.4678 | No |
| 12 | MRM3 | mitochondrial rRNA methyltransferase 3 [Source:HGNC Symbol;Acc:HGNC:18485] | 4806 | 0.087 | 0.4187 | No |
| 13 | ASMT | acetylserotonin O-methyltransferase [Source:HGNC Symbol;Acc:HGNC:750] | 5986 | -0.003 | 0.3277 | No |
| 14 | CMTR1 | cap methyltransferase 1 [Source:HGNC Symbol;Acc:HGNC:21077] | 7951 | -0.151 | 0.2019 | No |
| 15 | MRM1 | mitochondrial rRNA methyltransferase 1 [Source:HGNC Symbol;Acc:HGNC:26202] | 9114 | -0.241 | 0.1540 | No |
| 16 | FTSJ3 | FtsJ RNA 2'-O-methyltransferase 3 [Source:HGNC Symbol;Acc:HGNC:17136] | 9193 | -0.247 | 0.1913 | No |
| 17 | TRMT13 | tRNA methyltransferase 13 homolog [Source:HGNC Symbol;Acc:HGNC:25502] | 11792 | -0.548 | 0.0860 | No |
Table: GSEA details [plain text format]

  

Fig 2: GOMF\_O\_METHYLTRANSFERASE\_ACTIVITY      
 Blue-Pink O' Gram in the Space of the Analyzed GeneSet

  

Fig 3: GOMF\_O\_METHYLTRANSFERASE\_ACTIVITY: Random ES distribution      
 Gene set null distribution of ES for **GOMF\_O\_METHYLTRANSFERASE\_ACTIVITY**
